# Field investigation‐ and dietary metabarcoding‐based screening of arthropods that prey on primary tea pests

**DOI:** 10.1002/ece3.9060

**Published:** 2022-07-04

**Authors:** Tingbang Yang, Xuhao Song, Yang Zhong, Bin Wang, Caiquan Zhou

**Affiliations:** ^1^ Key Laboratory of Southwest China Wildlife Resources Conservation (Ministry of Education) China West Normal University Nanchong China; ^2^ Institute of Ecology China West Normal University Nanchong China; ^3^ School of Nuclear Technology and Chemistry & Biology Hubei University of Science and Technology Xianning China; ^4^ Hubei Engineering Research Center for Fragrant Plants Hubei University of Science and Technology Xianning China

**Keywords:** biological control, diet analysis, DNA metabarcoding, predatory arthropods, spiders, tea pests

## Abstract

Predatory natural enemies play key functional roles in biological control. Abundant predatory arthropod species have been recorded in tea plantation ecosystems. However, few studies have comprehensively evaluated the control effect of predatory arthropods on tea pests in the field. We performed a 1‐year field investigation and collected predatory arthropods and pests in the tea canopy. A total of 7931 predatory arthropod individuals were collected, and *Coleosoma blandum* (Araneae, Theridiidae) was the most abundant species in the studied tea plantation. The population dynamics between *C. blandum* and four main tea pest species (*Aleurocanthus spiniferus*, *Empoasca onukii*, *Ectropis grisescens*, and *Scopula subpunctaria*) were established using the individual number of predators and pests in each month. The results showed that *C. blandum* appeared to co‐occur in the tea canopy with *A. spiniferus*, *Em. onukii*, and *Ec. grisescens* in a longer period. The prey spectrum of *C. blandum* was further analyzed using DNA metabarcoding. Among prey species, *A. spiniferus*, *Em. onukii*, and *Ec. grisescens* were included, and the relative abundance and positive rates of target DNA fragments of *A. spiniferus* were greater than that of other two pests. Combined with the high dominance index of *C. blandum*, co‐occurrence between *C. blandum* and *A. spiniferus* in time and space and high positive rate and relative abundance of target DNA fragments of *A. spiniferus*, *C. blandum* was identified to prey on *A. spiniferus*, and *C. blandum* may be an important predator of *A. spiniferus*. Thus, *C. blandum* has potential as a biological control agent of *A. spiniferus* in an integrated pest management strategy.

## Graphical abstract

(1) *Coleosoma blandum* (Araneae, Theridiidae) was the most abundant species in the studied tea plantation and accounted for 34.80% of the total individual number of predatory arthropods; (2) *C. blandum* appeared to co‐occur in the tea canopy with three main tea pest species (*A. spiniferus*, *Em. onukii* and *Ec. grisescens*) in a longer period; (3) *A. spiniferus*, *Em. onukii* and *Ec. grisescens* were included in the prey spectrum of *C. blandum*, and the relative abundance and positive rates of target DNA fragments of *A. spiniferus* were greater than that of other two pests

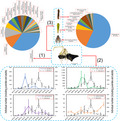

## 1 INTRODUCTION

*Camellia sinensis* is an important economic plant that is widely cultivated in many countries across Asia, Africa, Latin America, and Oceania (Cranham, 1966; Hazarika et al., 2009). Tea is one of the three major beverages (tea, cocoa, and coffee) consumed worldwide, and its production has become an important part of the national economy of many tea‐producing countries (Hazarika et al., 2009). However, many insect and mite pests breed in tea plantation ecosystems, which leads to reductions in the yield and quality of tea (Zhang & Tan, 2004). To reduce the economic losses caused by tea pests, a series of cultural, physical, genetic, biological, and chemical techniques have been applied as control measures (Hazarika et al., 2009). Among these techniques, chemical control (direct application of chemical pesticides to control pests) has been commonly applied due to its rapidly observed benefits. However, chemical control inevitably leads to many negative effects, such as pesticide residues, pest resistance, natural enemy mortality, secondary pest outbreaks, and environmental contamination (Baker et al., 2002; Hazarika et al., 2009; Lewis et al., 1997), with pesticide residue representing the main concern of consumers. Pesticide residues in tea directly affect the health of consumers and are also an important constraint factor in the tea trade (Feng et al., 2015). Therefore, identifying methods of reducing or eliminating chemical pesticide use in tea plantations has represented a primary focus of agricultural research (Hazarika et al., 2009).

Biological control in which natural enemies (predators, parasitoids, and pathogenic microorganisms) are used to control pests has become an essential component of integrated pest management (IPM; Giles et al., 2017). Predatory natural enemies control pest population numbers by killing or eating them; thus, they play an important role in biological control (Östman et al., 2003; Rendon et al., 2018). To date, many predatory natural enemies have been successfully used for agricultural pest control. For example, ladybird beetles (*Coccinella septempunctata*, *Harmonia axyridis*, and *Propylea japonica*) have been successfully used to control aphid pests (Arshad et al., 2017; Ouyang et al., 2012; Xue et al., 2009) while predatory mites (*Amblyseius swirskii*, *Phytoseiulus macropilis*, and *Neoseiulus californicus*) have been successfully used to control mite pests, thrips, whiteflies, etc. (Fonseca et al., 2020; Knapp et al., 2018; Oliveira et al., 2007; van Maanen et al., 2010). However, before using predators for pest control, the main predators of the target pests must be identified under field conditions (Yang, Liu, Yuan, Zhang, Peng, et al., 2017).

Many natural enemy species of the pests that inhabit tea plantation ecosystems have been identified, and these ecosystems provide favorable conditions for the protection and utilization of natural enemies for pest control (Ye et al., 2014). More than 1100 species of natural enemies (including predators, parasitoids, and pathogenic microorganisms) have been recorded in tea plantation ecosystems in China. Among them, predator species are the most abundant and account for 54.5% of the total number of natural enemy species (Ye et al., 2014; Zhang & Tan, 2004). Among these predators, most species are arthropods, with a few species belonging to insectivorous vertebrates. Although many predatory arthropods have been recorded in tea plantations, few studies have comprehensively evaluated the control effect of predators on tea pests in the field and the use of these predators for tea pest control.

At present, the ability of predators to control target pests under field conditions is mainly evaluated from three aspects: (1) Are predators present in large enough numbers? (2) Do predators and target pests come into contact in time and space? (3) Do the predators eat the target pests? For the first two questions, field investigations can be performed to determine the dominance of predators and temporal and spatial dynamic relationships between predators and pests (Dang et al., 2010; Ye et al., 2010). For the last question, a predator diet analysis can be performed to determine the prey composition of predators (Yang et al., 2021). In tea plantation ecosystems, the control of predators on tea pests is mostly evaluated based on the dominance of predators and temporal and spatial dynamic relationships between predators and pests. Many previous studies have identified a number of dominant predatory arthropod species that inhabit tea plantations, and many predatory arthropods appeared to co‐occur in the tea canopy with tea pests in time and space (Ke et al., 2011; Song et al., 2020; Zhang & Tan, 2004). However, few reports have detailed diet analyses of predators in tea plantations. Therefore, we sought to use a diet analysis method that could directly analyze the prey spectrum of predators in the field and combine field investigation to screen the predators of main tea pests.

Molecular gut content analysis is widely used to study predation (Rondoni et al., 2015). Conventional PCR and real‐time quantitative PCR are suitable for detecting predation by predators on a single prey or a few prey species based on prey‐specific primers (Yang, Liu, Yuan, Zhang, Li, et al., 2017; Yang et al., 2020). DNA metabarcoding can be used to analyze the prey composition of euryphagous predators based on prey‐universal primers (Tercel et al., 2021), and universal primers can be designed using the DNA barcodes of potential prey. The DNA fragments of prey remaining in the predator's gut or feces can be sequenced by next‐generation sequencing (NGS) technology using designed primers, and the results can then be matched with DNA barcodes from a public database or to a prey DNA library specifically designed for the study. To date, DNA metabarcoding has been successfully used for diet analysis of predators (Ingala et al., 2021; Lopes et al., 2020; Toju & Baba, 2018; Wang et al., 2022).

The cytochrome oxidase subunit I (COI) gene is widely used in DNA barcoding for species identification, especially in the taxonomic classification of insects, fish, and birds (Cheng et al., 2011). To date, large numbers of COI genes of various species have been deposited in public databases. Therefore, the COI gene has been successfully used as a barcode gene marker for predation detection; moreover, previous studies have shown that the COI gene can be effectively used in the diet analysis of predatory arthropods (Batuecas et al., 2021; Vasquez et al., 2021; Verdasca et al., 2021). Tea pests are mainly insects (Zhang & Tan, 2004), and many COI genes of tea pests have been uploaded to GenBank. Therefore, the present study chose the COI gene as the DNA barcode gene marker of tea pests.

The studied tea plantation was located at Chengjia town, Chengdu city, Sichuan Province, China. Our previous field investigation found that *Coleosoma blandum* (Araneae, Theridiidae) frequently appeared in the tea canopy of the studied tea plantation, and this species is widely distributed in tea plantations of China (Song et al., 2020). Therefore, our research questions focused mainly on: (1) is *C. blandum* the most dominant predatory arthropod in the studied tea plantation? (2) do *C. blandum* and main tea pests come into contact in time and space? (3) and whether *C. blandum* preys on the main tea pests in the studied tea plantation? Based on these questions, the present study focused mainly on three aspects: (1) a field investigation was performed to determine the dominant predatory arthropods and main tea pests and establish the temporal and spatial dynamic relationships between the dominant predatory arthropods and main tea pests; (2) DNA metabarcoding was used to analyze the prey spectra of dominant predatory arthropods; and (3) a comprehensive evaluation of dominant predatory arthropod predation on the main tea pests was performed and the main predator species of the main tea pests were screened.

## 2 MATERIALS AND METHODS

### 2.1 Collection and identification of specimens

**FIGURE 1:**
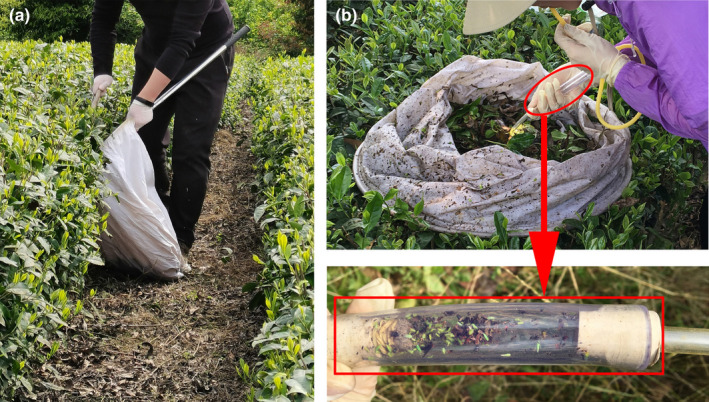
Sampling methods. (a) Beating the canopy of *Camellia sinensis*; (b) collecting samples with a homemade suction device

The study site was located at the tea plantation of Chengjia town, Chengdu city, Sichuan Province, China (103.37E; 30.19 N). *Camellia sinensis* is the main cash crop in this town. Approximately, 10 ha of a tea plantation was chosen for sampling. The studied tea plantation is an organic tea plantation, and the tea plants were cultivated in parallel rows approximately 20 m long and 1 m apart. The specimens were collected by the same person three times a month for at least 7 days in each period from May 2020 to April 2021. A total of 20 transects separated by at least 10 m were randomly chosen for each sampling event. The specimens were collected by a person who moved slowly along each transect while simultaneously beating the canopy of *C. sinensis* with a 0.5‐m wooden stick (2 cm in diameter) above an insect net (50 cm in diameter). After each transect was beaten, all arthropods in the insect net were collected by hand and by the use of a homemade suction device (Figure 1). The flying arthropods were collected first as they tended to fly away from the net. After collection, the predators were individually put into 1.5‐ml microcentrifuge tubes, and other arthropods were placed in plastic bottles (200 ml). All specimens were soaked with 100% ethanol and stored at −20°C. To avoid the impact of rain and insecticide on collection, the specimens were collected on dry days, and insecticide was not applied to the studied tea plantation during the sampling period. All specimens were identified from the reference keys and catalogs provided by Zhang and Tan (2004), Song et al. (2020), and the World Spider Catalog (2022). After identification, the individual numbers of each predator and pest species were counted.

### 2.2 Data analysis

The dominance of each predatory arthropod and pest species was calculated using the Berger–Parker index (Berger & Parker, 1970): *D* = *N*_*i*_/*N* (where *N*_*i*_ is the individual number of species *i* and *N* is the total number of predatory arthropods or pests). The dominant predatory arthropod species were determined by the Berger–Parker index of predators, and the main tea pest species were determined by the Berger–Parker index and damage characteristics of pests. To clarify the population dynamics between dominant predatory arthropods and the main tea pests, Microsoft Excel 2016 software (Microsoft) was used to generate a population dynamics diagram of dominant predatory arthropods and main tea pests based on the individual number of predatory arthropods and pests in each month.

### 2.3 DNA extraction

The dominant predatory arthropod species (*C. blandum*) was used for genomic DNA extraction. The genomic DNA of *C. blandum* was extracted individually. Due to the small body size of *C. blandum* (2–3 mm) and the extensive bifurcation of the spider gut (Foelix, 2011), the gut is not easily dissected from the surrounding tissues. Therefore, the whole spider body was used for DNA extraction (Toju & Baba, 2018). A total of 30 individuals (including juvenile and adult males and females) were randomly chosen and used for DNA extraction. To avoid contamination, each specimen was cleaned with ultrapure water before extraction. The specimens were then placed individually into 1.5‐ml microcentrifuge tubes. The genomic DNA of the whole predator body was extracted individually using the 2 × CTAB method as described by Vallet et al. (2008). Ultrapure water was used as a negative control for each extraction process. The DNA of each extraction was eluted in 50 μl of DNase‐free water. The quantity (Table S1) and quality (Figure S1) of the extracted DNA were measured using a NanoDrop ND‐1000 spectrophotometer (Thermo Fisher Scientific) and agarose gel electrophoresis, respectively. The DNA samples were stored at −20°C and later used for library preparation and sequencing.

### 2.4 Library preparation and sequencing

The forward primer mlCOIintF (GGWACWGGWTGAACWGTWTAYCCYCC) and reverse primer Fol‐degen‐rev (TANACYTCNGGRTGNCCRAARAAYCA; Krehenwinkel et al., 2017) were used to amplify prey DNA from the extracted DNA. The primers, which have been shown to successfully amplify a wide range of arthropods, amplified a 363‐bp amplicon located within the COI barcode region (Krehenwinkel et al., 2017). Sample‐specific 7‐bp barcodes (Table S2) were incorporated into the primers for multiplex sequencing. The individual DNA samples were amplified by a 2720 Thermal Cycler (Applied Biosystems) using the primers described above. Amplification was carried out in a final volume of 25 μl, with each tube containing 5 μl of Q5® High‐Fidelity GC buffer (5×), 0.25 μl of Q5® High‐Fidelity DNA Polymerase (5 U/μl, New England Biolabs), 5 μl of Q5® reaction buffer (5×), 2 μl (2.5 mM) of dNTPs, 2 μl of DNA template, 1 μl (10 μM) of each forward and reverse primer, and 8.75 μl of ddH_2_O. The thermal cycle consisted of an initial denaturation step at 98°C for 5 min, 27 cycles of denaturation at 98°C for 30 s, annealing at 50°C for 30 s, elongation at 72°C for 45 s, and a final extension at 72°C for 5 min. Each run contained a negative control (without DNA template). PCR products were purified with VAHTSTM DNA Clean Beads (Vazyme) and quantified using the Quant‐iT PicoGreen dsDNA Assay Kit (Invitrogen). The purification and quantification processes were performed according to the manufacturer's instructions. After the individual quantification step (Table S3), the PCR products were pooled in equimolar amounts, and then paired‐end 2 × 250‐bp sequencing was performed on the Illumina NovaSeq PE250 platform (Illumina) with NovaSeq 6000 SP Reagent Kit (500 cycles) (Illumina) at Shanghai Personal Biotechnology Co., Ltd (Shanghai, China).

### 2.5 Sequence analysis

All sequences were analyzed using QIIME2 (Version 2019.4; Bolyen et al., 2019) according to official tutorials (https://docs.qiime2.org/2019.4/tutorials/), with slight modifications. Briefly, raw sequencing reads that exactly matched the sample‐specific barcodes were assigned to respective samples and identified as valid sequences. The sequences were then merged, quality filtered, and dereplicated using the functions fastq_mergepairs, fastq_filter, and derep_fulllength in VSEARCH software, respectively (Rognes et al., 2016). After chimera detection, the remaining high‐quality sequences were clustered into operational taxonomic units (OTUs) at 97% sequence identity by UCLUST (Edgar, 2010). A representative sequence was selected from each OTU using default parameters. OTU taxonomical assignments were performed using the BROCC (https://github.com/kylebittinger/q2‐brocc#the‐brocc‐algorithm) against the NCBI‐nt database. An OTU table was further generated to record the relative abundance of each OTU in each sample and the taxonomy of the OTUs.

## 3 RESULTS

### 3.1 The dominant predatory arthropods

**FIGURE 2:**
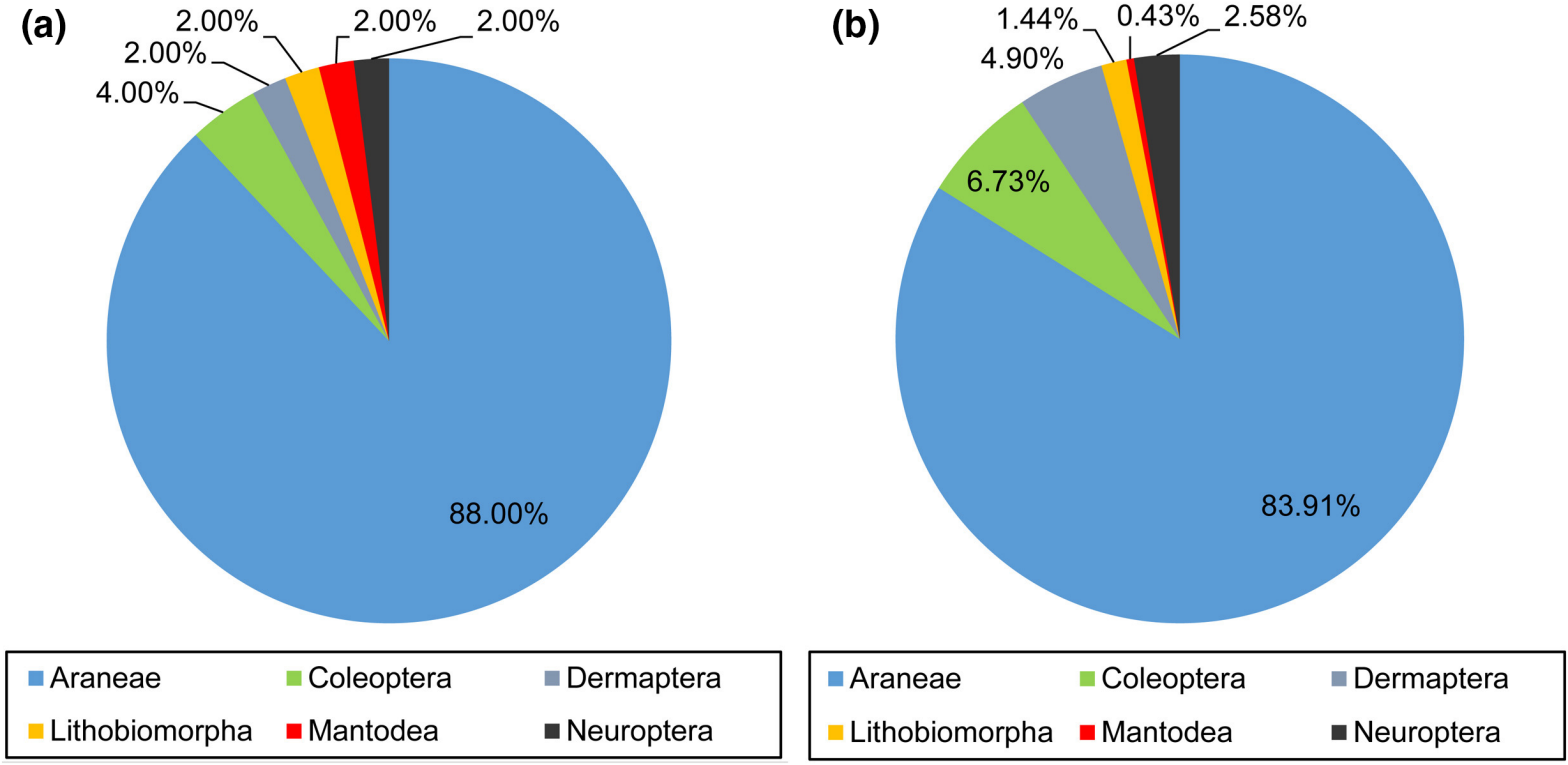
Statistics of the species number and individual number of predatory arthropods collected from the studied tea plantation at the order level. (a) Percentage of the species number; (b) percentage of the individual number

Through a 1‐year field investigation, a total of 7931 individuals of predatory arthropods were collected, and 50 species belonging to 19 families and 6 orders were identified (Table 1). Among them, Araneae species were the most abundant (44 species), accounting for 88.00% of the total number of predatory arthropod species (Figure 2a). In addition, spiders were the most abundant arthropods, accounting for 83.91% of the total individual number of predatory arthropods (Figure 2b). Among these spiders, *C. blandum* (Figure S2) was present in large numbers in the studied tea plantation, accounting for 34.80% of the total individual number of predatory arthropods (Table 1).

**TABLE 1:** Dominance of predatory arthropods collected from the studied tea plantation. Both juveniles and adults of predatory arthropods were used to calculate dominance

Class	Order	Family	Species	Individual number	Dominance, %
Arachnida	Araneae	Agelenidae	*Agelena* sp.	10	0.13
Arachnida	Araneae	Araneidae	*Araneus ejusmodi*	43	0.54
Arachnida	Araneae	Araneidae	*Araneus pentagrammicus*	18	0.23
Arachnida	Araneae	Araneidae	*Cyclosa argenteoalba*	22	0.28
Arachnida	Araneae	Araneidae	*Cyrtarachne nagasakiensis*	1	0.01
Arachnida	Araneae	Araneidae	*Eriovixia cavaleriei*	27	0.34
Arachnida	Araneae	Araneidae	*Neoscona scylla*	5	0.06
Arachnida	Araneae	Araneidae	*Neoscona vigilans*	35	0.44
Arachnida	Araneae	Clubionidae	*Clubiona deletrix*	1	0.01
Arachnida	Araneae	Hahniidae	*Hahnia thorntoni*	86	1.08
Arachnida	Araneae	Linyphiidae	*Erigone prominens*	2	0.03
Arachnida	Araneae	Linyphiidae	*Hylyphantes graminicola*	10	0.13
Arachnida	Araneae	Linyphiidae	*Neriene cavaleriei*	1	0.01
Arachnida	Araneae	Linyphiidae	*Ummeliata feminea*	6	0.08
Arachnida	Araneae	Linyphiidae	*Ummeliata insecticeps*	2	0.03
Arachnida	Araneae	Lycosidae	*Ovia alboannulata*	871	10.98
Arachnida	Araneae	Oxyopidae	*Oxyopes* sp.	42	0.53
Arachnida	Araneae	Philodromidae	*Philodromus subaureolus*	5	0.06
Arachnida	Araneae	Pisauridae	*Dolomedes* sp.	174	2.19
Arachnida	Araneae	Salticidae	*Bristowia heterospinosa*	258	3.25
Arachnida	Araneae	Salticidae	*Evarcha albaria*	264	3.33
Arachnida	Araneae	Salticidae	*Myrmarachne gisti*	39	0.49
Arachnida	Araneae	Salticidae	*Orienticius vulpes*	8	0.10
Arachnida	Araneae	Salticidae	*Phintella bifurcilinea*	9	0.11
Arachnida	Araneae	Salticidae	*Sibianor* sp.	148	1.87
Arachnida	Araneae	Salticidae	*Thiania cavaleriei*	20	0.25
Arachnida	Araneae	Tetragnathidae	*Tetragnatha maxillosa*	249	3.14
Arachnida	Araneae	Theridiidae	*Chrosiothes sudabides*	13	0.16
Arachnida	Araneae	Theridiidae	*Chrysso* sp.	2	0.03
Arachnida	Araneae	Theridiidae	*Coleosoma blandum*	2760	34.80
Arachnida	Araneae	Theridiidae	*Coleosoma floridanum*	87	1.10
Arachnida	Araneae	Theridiidae	*Coleosoma octomaculatum*	57	0.72
Arachnida	Araneae	Theridiidae	*Meotipa spiniventris*	10	0.13
Arachnida	Araneae	Theridiidae	*Meotipa vesiculosa*	8	0.10
Arachnida	Araneae	Theridiidae	*Paidiscura subpallens*	199	2.51
Arachnida	Araneae	Theridiidae	*Phycosoma sinica*	77	0.97
Arachnida	Araneae	Theridiidae	*Platnickina mneon*	116	1.46
Arachnida	Araneae	Theridiidae	*Theridion submirabile*	1	0.01
Arachnida	Araneae	Thomisidae	*Ebrechtella tricuspidata*	37	0.47
Arachnida	Araneae	Thomisidae	*Oxytate* sp.	5	0.06
Arachnida	Araneae	Thomisidae	*Thomisus eminulus*	6	0.08
Arachnida	Araneae	Thomisidae	*Xysticus croceus*	755	9.52
Arachnida	Araneae	Thomisidae	*Xysticus kurilensis*	8	0.10
Arachnida	Araneae	Trachelidae	*Trachelas sinensis*	158	1.99
Chilopoda	Lithobiomorpha	Lithobiidae	*Eupolybothrus* sp.	114	1.44
Insecta	Coleoptera	Coccinellidae	*Chilocorus kuwanae*	274	3.45
Insecta	Coleoptera	Coccinellidae	*Serangium japonicum*	260	3.28
Insecta	Dermaptera	Forficulidae	*Forficula* sp.	389	4.90
Insecta	Mantodea	Mantidae	*Statilia maculata*	34	0.43
Insecta	Neuroptera	Hemerobiidae	Unknown	205	2.58

### 3.2 Population dynamics between dominant predatory arthropods and main tea pests

**FIGURE 3:**
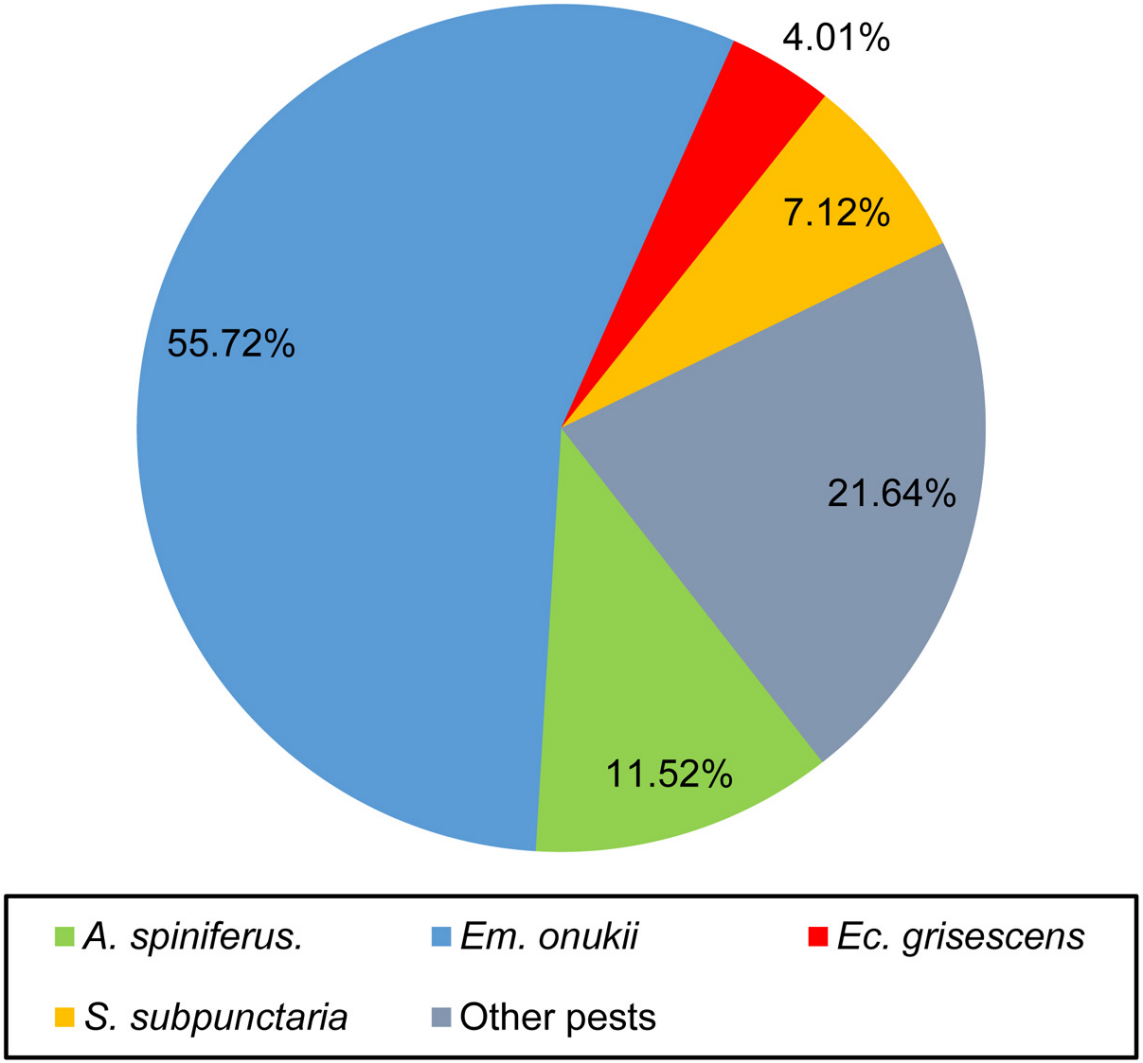
Dominance of four main tea pest species collected from the studied tea plantation. The dominance is shown in the pie chart as a percentage

**FIGURE 4:**
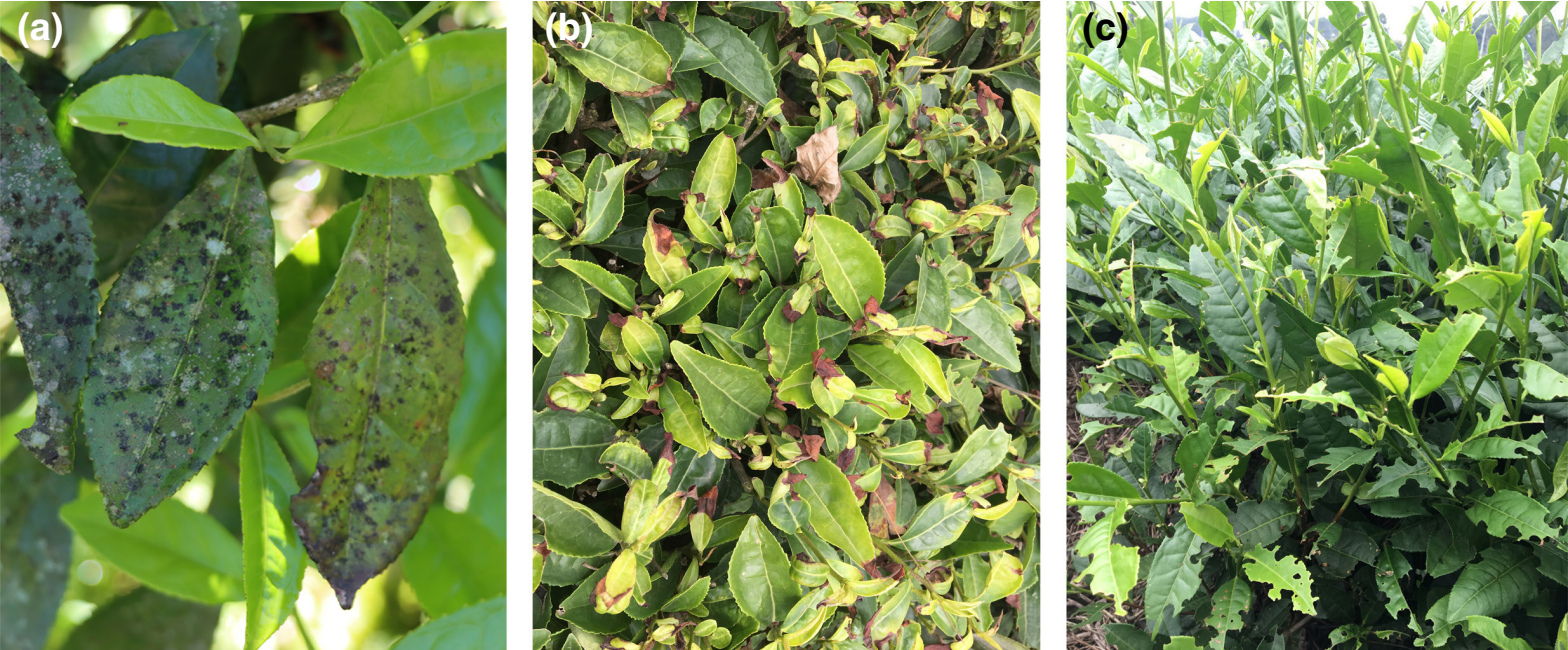
Damage characteristics of four main tea pest species when they occurred in large numbers. (a) Damage characteristics of *Aleurocanthus spiniferus*, with the damaged leaves appearing mildew (nymphs suck juices out of the tea‐leaf, honeydew secreted by nymphs can induce mold parasitism); (b) damage characteristics of *Empoasca onukii*, with the damaged leaves appearing scorched; (c) damage characteristics of *Ectropis grisescens* and *Scopula subpunctaria*, with the damaged leaves appearing incomplete and showing nicks and holes

**FIGURE 5:**
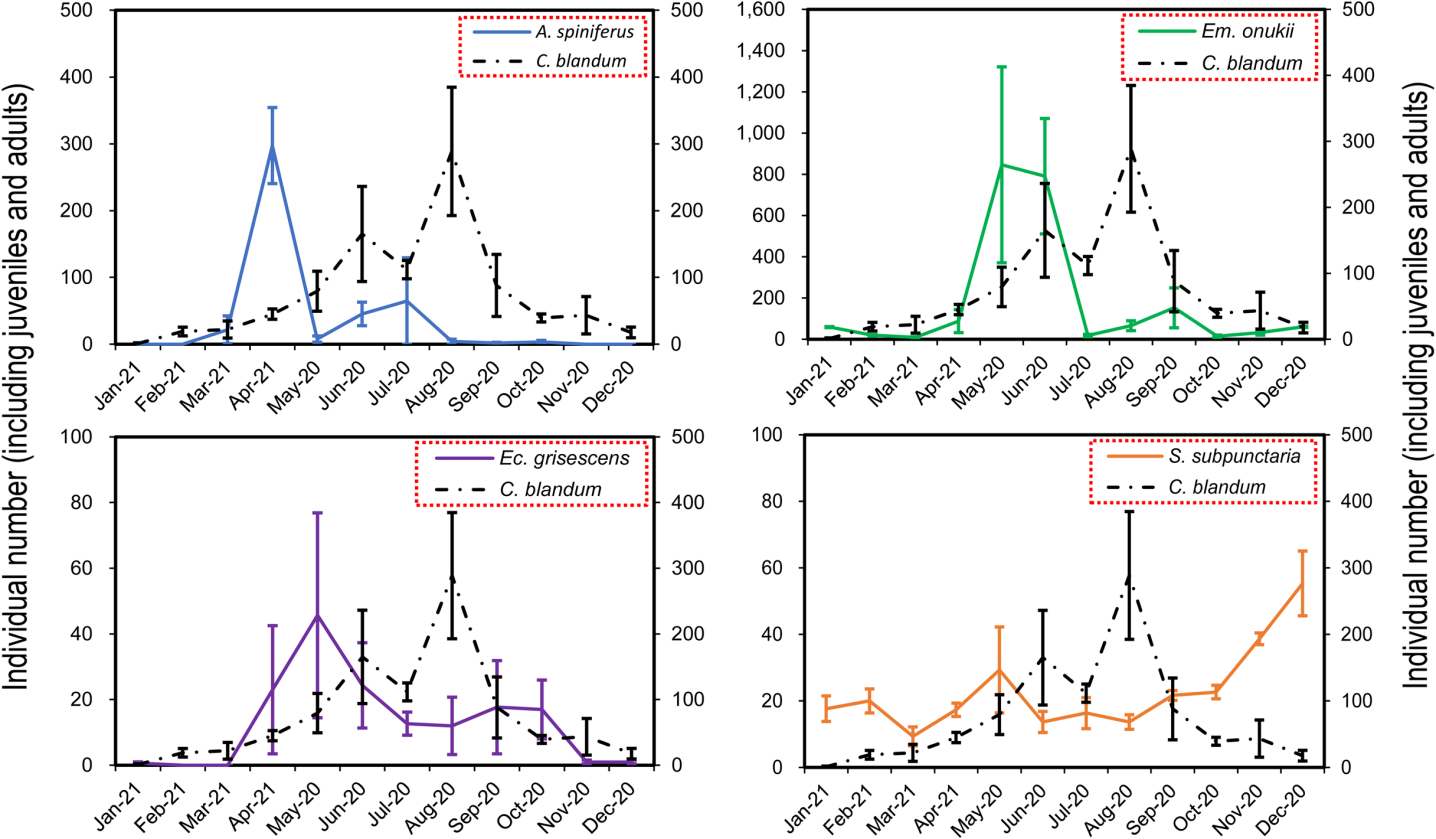
Population dynamics between dominant predator species (*Coleosoma blandum*) and four main tea pest species (*Aleurocanthus spiniferus*, *Empoasca onukii*, *Ectropis grisescens* and *Scopula subpunctaria*) in the studied tea plantation. Values are presented as the mean ± *SE* (*N* = 3)

In addition to predatory arthropods, a total of 21,504 other arthropods were collected in the studied tea plantation, and 11 orders were identified (Table S4). Except for some neutral arthropods (including all collembolans and a few insects [Diptera and Formicidae]), the other arthropods were tea pests. According to the dominance (Figure 3) and damage characteristics of the pests (Figure 4), four main tea pest species (*Aleurocanthus spiniferus* (Hemiptera, Aleyrodidae; Figure S3a), *Empoasca onukii* (Hemiptera, Cicadellidae; Figure S3b), *Ectropis grisescens* (Lepidoptera, Geometridae; Figure S3c), and *Scopula subpunctaria* (Lepidoptera, Geometridae; Figure S3d) were confirmed in the studied tea plantation. We established the population dynamics between four main tea pest species and dominant predator species (*C. blandum*) using the individual number of predators and pests in each month. As shown in Figure 5, the dominant predator species (*C. blandum*) appeared mainly from March to November, and three main tea pest species (*A. spiniferus*, *Em. onukii*, and *Ec. grisescens*) appeared mainly from March to August, April to October, and March to November, respectively. These results showed that *C. blandum* appeared to co‐occur in the tea canopy with *A. spiniferus*, *Em. onukii*, and *Ec. grisescens* in a longer period. *S. subpunctaria* appeared mainly from November to December, while the population number of *C. blandum* was relatively low in this period.

### 3.3 Prey spectra of *C. blandum*

A total of 2,903,857 raw sequences were obtained after 30 DNA samples were sequenced using the Illumina NovaSeq PE250 platform. A total of 2,759,993 high‐quality sequences were obtained after the sequences were merged and filtered and chimeras were removed (Table S5). For each DNA sample sequence, the high‐quality sequences were clustered into OTUs at 97% sequence identity. The representative sequence from each OTU was identified using the BROCC against the NCBI‐nt database. The results showed that 8340 sequences were assigned to prey sequences, which accounted for 0.3% of the total sequences. A total of 42 OTUs were obtained after the 8340 prey sequences were annotated. Among them, 42, 41, 31, and 14 OTUs were identified to the order, family, genus, and species levels, respectively, which accounted for 100.0%, 97.6%, 73.8%, and 33.3% of the total OTU number, respectively. A total of 4 classes, 11 orders, 33 families, 31 genera, and 14 species of prey were identified (Table S6). Three main tea pest species (*A. spiniferus*, *Em. onukii*, and *Ec. grisescens*) were included among the prey species (Table S6), and the number of corresponding prey sequences was 1589 for *A. spiniferus*, 14 for *Em. onukii* and 3 for *Ec. grisescens*, which accounted for 19.05%, 0.17%, and 0.04% of the total number of prey sequences, respectively (Table 2). In addition, we calculated the positive rates of target DNA fragments of pests remaining in the predator's gut. As shown in Table 2, among the 30 DNA samples, the positive rates of target DNA fragments of the three main tea pests remaining in the gut of *C. blandum* were 26.7% for *A. spiniferus*, 10.0% for *Em. onukii*, and 3.3% for *Ec. grisescens*.

**TABLE 2:** Positive rate and relative abundance of the target DNA fragments of four main tea pests. Thirty DNA samples extracted from *Coleosoma blandum* were sequenced, and 8340 prey sequences were annotated

Species	Number of tested spiders	Number of positive spiders	Positive rate (%)	Number of sequences	Relative abundance of sequences (%)
*Aleurocanthus spiniferus*	30	8	26.7	1589	19.05
*Empoasca onukii*	30	3	10.0	14	0.17
*Ectropis grisescens*	30	1	3.3	3	0.04
*Scopula subpunctaria*	30	0	0	0	0

## 4 DISCUSSION

*A. spiniferus*, *Em. onukii*, *Ec. grisescens*, and *S. subpunctaria* are the main tea pests that are widely distributed in many tea‐producing regions in China (Zhang & Tan, 2004). The quality and yield of tea are seriously reduced when these pests occur in large numbers. Therefore, identifying predators for the control of these tea pests is vital. Abundant predatory natural enemies inhabit in tea plantation ecosystems (Ye et al., 2014). To screen the main predators of the main tea pests, we performed a 1‐year field investigation in the studied tea plantation, collected predators and tea pests, and then analyzed the prey spectra of the dominant predator species (*C. blandum*) using DNA metabarcoding. Finally, the control efficiency of predators on target pests was comprehensively evaluated.

Chen et al. (2004) performed a comprehensive investigation of predator species in Chinese tea plantations. The results showed that many spider species were recorded in tea plantations, and spiders were the most species‐rich when compared to other predator taxa. In addition, the relative abundance of spiders was also higher than that of other predator taxa, accounting for 65.0%–97.8% of the total number of predators. Our results showed that among predatory arthropods, spiders were the most species‐rich and showed the greatest relative abundance in the studied tea plantation (Figure 2). Among spider species, *C. blandum* was the most dominant species in the studied tea plantation, with a dominance index of 34.80% (Table 1). This species is widely distributed in tea plantations in Fujian, Guangdong, Yunnan, and Zhejiang of China and recorded as the dominant species in tea plantations of Guangdong and Zhejiang provinces (Song et al., 2020). Therefore, the predatory behavior of *C. blandum* on target pests should be further investigated.

The temporal and spatial co‐occurrence between predators and pests are often used as important indices to evaluate the control effect of predators on pests (Yang, Liu, Yuan, Zhang, Peng, et al., 2017). The temporal and spatial co‐occurrence between predators and pests indicates the potential of predators as biological control agents for pests (Liu et al., 2000). Our results showed that the dominant spider species (*C. blandum*) appeared to co‐occur in the tea canopy with three main tea pest species (*A. spiniferus*, *Em. onukii* and *Ec. grisescens*) in a longer period (Figure 5). In terms of spatial co‐occurrence, *C. blandum* was spatially co‐occurring with four main tea pest species in the studied tea plantation because they were collected in the tea canopy (Yang, Liu, Yuan, Zhang, Peng, et al., 2017). The spatial co‐occurrence between predators and pests indicates that predators and pests present a greater probability of encounter, which reduces the time for predators to search for prey and increases the opportunity for predation (Chen et al., 2002).

To confirm whether *C. blandum* prey on target pests in the field, we analyzed the prey spectrum of *C. blandum* collected from the studied tea plantation. The genomic DNA extracted from whole spider body was sequenced using NGS technology based on prey‐universal primers (mlCOIintF/Fol‐degen‐rev; Krehenwinkel et al., 2017). Universal primer pairs (mlCOIintF/Fol‐degen‐rev) can amplify the COI gene in the prey remains within the spider's gut as well as in the spider itself. Similar to the results of Piñol et al. (2014) and Yang et al. (2021), the sequence annotation showed that most of the sequences belonged to the predator itself, which accounted for 90.9% of the total sequences. In addition, nonprey sequences (including fungi, Chordata, aquatic taxa (Cnidaria, Rotifera, Bacillariophyta, Phaeophyta, Rhodophyta, and some aquatic arthropods) and soil‐dwelling taxa (Annelida)) were found in the sequencing results, which accounted for 1.6% of the total sequences. They were likely introduced during the library preparation and sequencing processes (Salter et al., 2014; Weiss et al., 2014) because PCR can amplify minute quantities of contaminant DNA due to the high sensitivity of the method (Drake et al., 2022). Therefore, the sequencing results need to be interpreted appropriately and the predator and nonprey sequences should be removed in the dietary metabarcoding (Drake et al., 2022; Yang et al., 2021). In order to remove nonprey sequences, we considered the dietary characteristics of the spider; that is, it usually feeds on arthropods, especially insects and collembolans (Foelix, 2011; Nyffeler & Birkhofer, 2017). Our results showed that some sequences were not identified to the class level, which accounted for 7.1% of the total sequences. This is a common problem in dietary metabarcoding because sequence identification relies on the inclusion of prey species barcoding genes in public databases (Piñol et al., 2014). Therefore, to improve the ability to fully identify prey sequences from predator guts, further work needs to be carried out on the barcoding of organisms found in the studied tea plantations.

A total of 8340 sequences were used to annotate the prey spectrum of *C. blandum*. Abundant OTUs were obtained from these sequences, and most OTUs were identified to the genus level (accounting for 73.8% of the total OTU number). A total of 4 classes, 11 orders, 33 families, 31 genera, and 14 species of prey were identified, which is consistent with the euryphagous characteristics of spiders (Foelix, 2011). The prey spectrum analysis indicated that *C. blandum* mainly preys on small arthropods, which may be related to its small body size. A few arthropods with relatively large body sizes in adult stage were also included in the prey spectrum, such as Lepidoptera, Orthoptera, and Lithobiomorpha. We hypothesize that *C. blandum* probably prey on the juveniles of these prey. However, the life stage of prey cannot be identified by the present method because the primers were not specific to any life stage (Yang, Liu, Yuan, Zhang, Peng, et al., 2017). Similar to the results of Yang et al. (2021), a few spider species (*Neoleptoneta* sp., *Pardosa* sp., and *Theridion* sp.; Table S6) were included in the prey spectrum of *C. blandum*, thus indicating intraguild predation (Michalko et al., 2021). Intraguild predation is a common phenomenon observed in diet analyses of spiders (Saqib et al., 2021; Yang et al., 2021), and it is likely an adaptive strategy that helps spiders address energy limitations caused by scarce prey (Haghani et al., 2019; Michalko et al., 2021; Wise, 1995). Among the prey species, most included tea pests, and the three main tea pest species were found in the prey spectrum of *C. blandum* (Table S6). The relative abundance of target DNA fragments was 19.05% for *A. spiniferus*, 0.17% for *Em. onukii*, and 0.04% for *Ec. grisescens* (Table 2). In addition, the positive target DNA fragment rate of the three main tea pests remaining in the gut of *C. blandum* was 26.7% for *A. spiniferus*, 10.0% for *Em. onukii*, and 3.3% for *Ec. grisescens*, respectively (Table 2). The relatively high positive rate and relative abundance of the target DNA fragments predicted that *C. blandum* frequently preys on *A. spiniferus*.

Krehenwinkel et al. (2017) showed that the universal primer pair (mlCOIintF/Fol‐degen‐rev) could amplify the COI gene of many arthropod species, especially those belonging to Acari, Araneae, Coleoptera, Collembola, Diptera, Hemiptera, Lepidoptera, Myriapoda, Orthoptera, and Psocoptera. These arthropods were generally consistent with those collected from the studied tea plantation (Table S4). Our results also showed that universal primer pair (mlCOIintF/Fol‐degen‐rev) was effectively used in this study, which obtained a broad prey composition after 30 DNA samples were sequenced. In addition, the diversity of prey from the spider gut was consistent with the diversity of potential prey from the studied tea plantation (Table S4). However, DNA metabarcoding could not quantify the predation of predators on target pests. To date, effective methods are not available for quantifying the predation of predatory arthropods under field conditions. Therefore, additional work needs to be carried out to identify an effective method for quantifying predation to obtain more comprehensive evaluation indices. In any case, the high dominance index of *C. blandum*, co‐occurrence between *C. blandum* and *A. spiniferus* in time and space and high positive rate and relative abundance of the target DNA fragments of *A. spiniferus* indicated that *C. blandum* preys on *A. spiniferus*, and *C. blandum* may be an important predator of *A. spiniferus*. Thus, *C. blandum* has potential as a biological control agent of *A. spiniferus* in an IPM strategy.

## 5 CONCLUSIONS

In the present study, a comprehensive evaluation of dominant predatory arthropod predation on the main tea pests was performed and the main predator species of the main tea pests were screened. The results showed that (1) *C. blandum* (Araneae, Theridiidae) was the most abundant species in the studied tea plantation and accounted for 34.80% of the total individual number of predatory arthropods; (2) *C. blandum* appeared to co‐occur in the tea canopy with three main tea pest species (*A. spiniferus*, *Em. onukii*, and *Ec. grisescens*) in a longer period; (3) *A. spiniferus*, *Em. onukii*, and *Ec. grisescens* were included in the prey spectrum of *C. blandum*, and the relative abundance and positive rates of target DNA fragments of *A. spiniferus* were greater than that of other two pests; (4) *C. blandum* has potential as a biological control agent of *A. spiniferus* in an IPM strategy.

## AUTHOR CONTRIBUTIONS

**Tingbang Yang:** Conceptualization (lead); data curation (equal); formal analysis (equal); funding acquisition (equal); investigation (equal); methodology (equal); project administration (equal); supervision (equal); writing – original draft (equal); writing – review and editing (lead). **Xuhao Song:** Data curation (equal); formal analysis (equal); investigation (equal); methodology (equal); software (equal); supervision (equal); writing – original draft (equal); writing – review and editing (equal). **Yang Zhong:** Formal analysis (equal); investigation (equal); writing – original draft (equal). **Bin Wang:** Formal analysis (equal); software (equal); writing – original draft (equal). **Caiquan Zhou:** Project administration (equal); supervision (equal); writing – original draft (equal).

## CONFLICT OF INTEREST

The authors declare no competing or financial interests.

## Supporting information


Appendix S1.

## Data Availability

Raw sequences are available online on Dryad repository (https://doi.org/10.5061/dryad.rr4xgxdb2) and NCBI databases (https://www.ncbi.nlm.nih.gov/), the associated BioProject, SRA, and Bio‐Sample numbers are PRJNA842855, SRR19427103, and SAMN28688760, respectively.
