# Supplementary material for: Field investigation‐ and dietary metabarcoding‐based screening of arthropods that prey on primary tea pests
Source: Ecol Evol. 2022 Jul 4;12(7):e9060. doi: 10.1002/ece3.9060 (PMC9251880; doi:10.1002/ece3.9060)
Supplement: Supplementary file 1 — Appendix S1. [file ECE3-12-e9060-s001.docx]

**Supplementary material:**

**“Field investigation- and dietary metabarcoding-based screening of arthropods that prey on primary tea pests”**

Tingbang Yang^1,2^, Xuhao Song^1,2^, Yang Zhong^3,4^, Bin Wang^1,2^, Caiquan Zhou^1,2^

^1^Key Laboratory of Southwest China Wildlife Resources Conservation (Ministry of Education), China West Normal University, Nanchong, Sichuan, China

^2^Institute of Ecology, China West Normal University, Nanchong, Sichuan, China

^3^School of Nuclear Technology and Chemistry & Biology, Hubei University of Science and Technology, Xianning, Hubei, China

^4^Hubei Engineering Research Center for Fragrant Plants, Hubei University of Science and Technology, Xianning, Hubei, China

**Supplementary Figures**


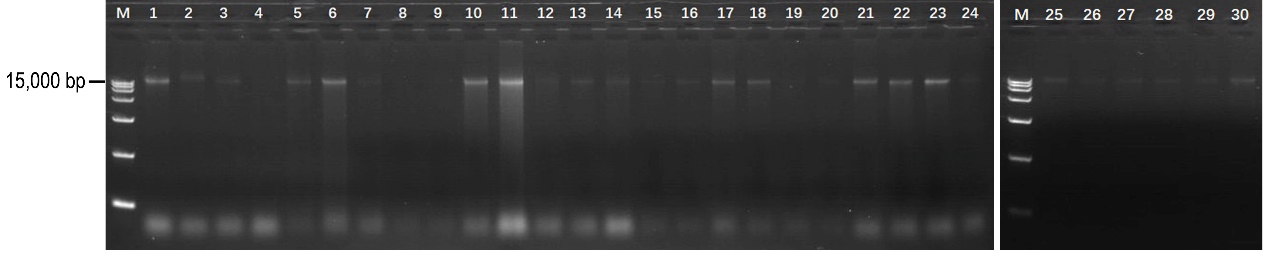


**Figure S1** Agarose gel electrophoresis of 30 DNA samples extracted from *C. blandum*. Cropped gels are merged and displayed (the full length of each uncropped gel is 11.5 cm). M: DNA marker (DL15,000 DNA Marker-Takara)


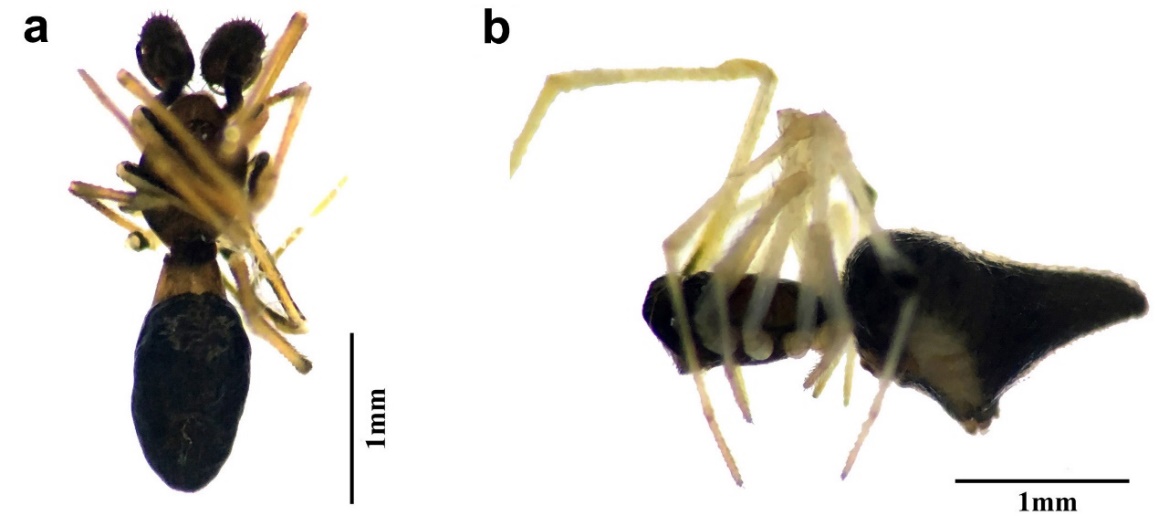


**Figure S2** Morphology of *C. blandum*. (a) Dorsal view; (b) Lateral view


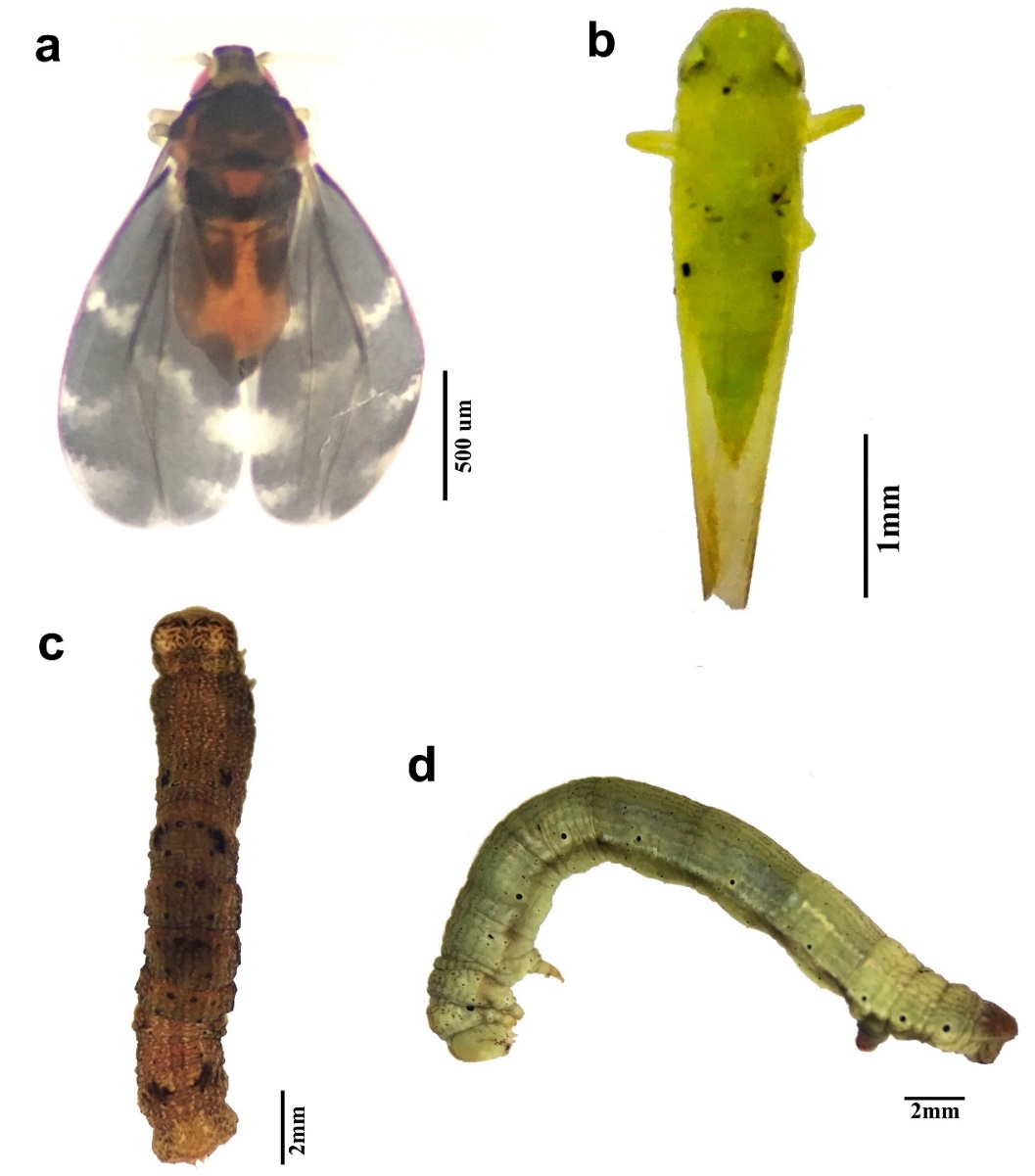


**Figure S3** Morphology of four main tea pests. (a) *Aleurocanthus spiniferus* (adult); (b) *Empoasca onukii* (adult); (c) *Ectropis grisescens* (larva); (d) *Scopula subpunctaria* (larva)

**Supplementary Tables**

**Table S1** Quantity of extracted DNA from *C. blandum*. The quantity was measured using a NanoDrop ND-1000 spectrophotometer

| Sample ID | Sample type | Volume (μl) | Concentration (ng/μl) | OD260/280 | OD260/230 |
| --- | --- | --- | --- | --- | --- |
| COB_01 | DNA | 50 | 109.20 | 1.94 | 1.46 |
| COB_02 | DNA | 50 | 70.40 | 1.95 | 1.54 |
| COB_03 | DNA | 50 | 80.00 | 1.94 | 1.42 |
| COB_04 | DNA | 50 | 85.90 | 2.00 | 1.62 |
| COB_05 | DNA | 50 | 29.00 | 2.09 | 1.17 |
| COB_06 | DNA | 50 | 26.80 | 1.99 | 0.28 |
| COB_07 | DNA | 50 | 51.00 | 1.97 | 1.38 |
| COB_08 | DNA | 50 | 24.50 | 1.93 | 1.01 |
| COB_09 | DNA | 50 | 34.50 | 1.88 | 1.18 |
| COB_10 | DNA | 50 | 55.40 | 1.95 | 1.47 |
| COB_11 | DNA | 50 | 68.00 | 1.92 | 1.09 |
| COB_12 | DNA | 50 | 81.50 | 1.98 | 1.66 |
| COB_13 | DNA | 50 | 70.10 | 1.99 | 1.62 |
| COB_14 | DNA | 50 | 90.60 | 1.91 | 1.63 |
| COB_15 | DNA | 50 | 31.90 | 1.93 | 1.28 |
| COB_16 | DNA | 50 | 37.50 | 1.92 | 1.35 |
| COB_17 | DNA | 50 | 29.90 | 1.90 | 1.23 |
| COB_18 | DNA | 50 | 39.40 | 1.89 | 1.34 |
| COB_19 | DNA | 50 | 43.70 | 1.92 | 1.28 |
| COB_20 | DNA | 50 | 32.70 | 1.94 | 1.19 |
| COB_21 | DNA | 50 | 71.00 | 1.90 | 1.28 |
| COB_22 | DNA | 50 | 77.30 | 1.93 | 1.52 |
| COB_23 | DNA | 50 | 96.70 | 1.95 | 1.56 |
| COB_24 | DNA | 50 | 55.80 | 1.99 | 1.50 |
| COB_25 | DNA | 50 | 28.60 | 1.99 | 1.08 |
| COB_26 | DNA | 50 | 14.50 | 2.10 | 1.32 |
| COB_27 | DNA | 50 | 28.30 | 1.99 | 1.05 |
| COB_28 | DNA | 50 | 24.40 | 1.94 | 1.13 |
| COB_29 | DNA | 50 | 24.80 | 2.01 | 1.10 |
| COB_30 | DNA | 50 | 24.40 | 1.92 | 1.05 |

**Table S2** Sample-specific 7-bp barcodes

| Sample ID | Sample type | Barcode |
| --- | --- | --- |
| COB_01 | DNA | CTTGAGT |
| COB_02 | DNA | CTCACGA |
| COB_03 | DNA | CTCAGAC |
| COB_04 | DNA | CTCTCAG |
| COB_05 | DNA | CTCTGTA |
| COB_06 | DNA | CTGATGT |
| COB_07 | DNA | CTGTAGA |
| COB_08 | DNA | GAACACT |
| COB_09 | DNA | GATCTCA |
| COB_10 | DNA | GACATCT |
| COB_11 | DNA | GACAGTA |
| COB_12 | DNA | GACTACA |
| COB_13 | DNA | GACTCAC |
| COB_14 | DNA | GAGTCGT |
| COB_15 | DNA | GTACAGA |
| COB_16 | DNA | GTAGTCT |
| COB_17 | DNA | GTCACAT |
| COB_18 | DNA | GTCTAGT |
| COB_19 | DNA | GTCGTGA |
| COB_20 | DNA | GTGAACA |
| COB_21 | DNA | GTGACTC |
| COB_22 | DNA | GTGTCAA |
| COB_23 | DNA | AACTGTC |
| COB_24 | DNA | AACGTGT |
| COB_25 | DNA | CTTGAGT |
| COB_26 | DNA | CTCACGA |
| COB_27 | DNA | CTCAGAC |
| COB_28 | DNA | CTCTCAG |
| COB_29 | DNA | CTCTGTA |
| COB_30 | DNA | CTGATGT |

**Table S3** Quantity of PCR products. The quantity was measured using a Quant-iT PicoGreen dsDNA Assay Kit (Invitrogen, USA)

| Sample ID | Amplification region | Fragment size (bp) | Concentration (ng/μl) |
| --- | --- | --- | --- |
| COB_01 | COI | 363 | 19.02 |
| COB_02 | COI | 363 | 16.01 |
| COB_03 | COI | 363 | 12.55 |
| COB_04 | COI | 363 | 8.07 |
| COB_05 | COI | 363 | 14.84 |
| COB_06 | COI | 363 | 10.62 |
| COB_07 | COI | 363 | 9.85 |
| COB_08 | COI | 363 | 5.09 |
| COB_09 | COI | 363 | 11.06 |
| COB_10 | COI | 363 | 13.52 |
| COB_11 | COI | 363 | 14.44 |
| COB_12 | COI | 363 | 16.10 |
| COB_13 | COI | 363 | 11.52 |
| COB_14 | COI | 363 | 9.80 |
| COB_15 | COI | 363 | 7.83 |
| COB_16 | COI | 363 | 12.14 |
| COB_17 | COI | 363 | 12.99 |
| COB_18 | COI | 363 | 7.79 |
| COB_19 | COI | 363 | 6.61 |
| COB_20 | COI | 363 | 9.10 |
| COB_21 | COI | 363 | 8.72 |
| COB_22 | COI | 363 | 9.66 |
| COB_23 | COI | 363 | 12.36 |
| COB_24 | COI | 363 | 8.18 |
| COB_25 | COI | 363 | 4.61 |
| COB_26 | COI | 363 | 4.08 |
| COB_27 | COI | 363 | 4.86 |
| COB_28 | COI | 363 | 4.07 |
| COB_29 | COI | 363 | 7.46 |
| COB_30 | COI | 363 | 19.35 |

**Table S4** Arthropods collected from the studied tea plantation (not including predatory arthropods)

| Class | Order | Family | Species | Individual number |
| --- | --- | --- | --- | --- |
| Arachnida | Acarina | unknown | unknown | 191 |
| Collembola | Entomobryomorpha | Entomobryidae | unknown | 1244 |
| Collembola | unknown | unknown | unknown | 4228 |
| Insecta | Blattodea | Blattellidae | unknown | 10 |
| Insecta | Coleoptera | Chrysomelidae | unknown | 5 |
| Insecta | Coleoptera | Curculionidae | unknown | 5 |
| Insecta | Coleoptera | Meloidae | *Mylabris cichorii* | 1 |
| Insecta | Coleoptera | Nitidulidae | unknown | 12 |
| Insecta | Coleoptera | Scarabaeidae | unknown | 2 |
| Insecta | Coleoptera | unknown | unknown | 207 |
| Insecta | Corrodentia | unknown | unknown | 57 |
| Insecta | Diptera | Chloropidae | *Chlorops oryzae* | 6 |
| Insecta | Diptera | Chloropidae | *Elachiptera* sp. | 12 |
| Insecta | Diptera | Chloropidae | unknown | 2 |
| Insecta | Diptera | Drosophilidae | *Drosophila* sp. | 229 |
| Insecta | Diptera | Drosophilidae | *Drosophila triauraria* | 8 |
| Insecta | Diptera | Drosophilidae | *Microdrosophila* sp. | 24 |
| Insecta | Diptera | Drosophilidae | *Scaptomyza pallida* | 2092 |
| Insecta | Diptera | unknown | unknown | 1158 |
| Insecta | Hemiptera | Aleyrodidae | *Aleurocanthus spiniferus* | 1337 |
| Insecta | Hemiptera | Aphididae | unknown | 250 |
| Insecta | Hemiptera | Cicadellidae | *Empoasca onukii* | 6468 |
| Insecta | Hemiptera | Cicadellidae | unknown | 66 |
| Insecta | Hemiptera | Coccidae | unknown | 13 |
| Insecta | Hemiptera | Flatidae | unknown | 2 |
| Insecta | Hemiptera | Margarodidae | unknown | 25 |
| Insecta | Hemiptera | Pentatomidae | *Eysarcoris guttiger* | 102 |
| Insecta | Hemiptera | Pentatomidae | unknown | 6 |
| Insecta | Hemiptera | unknown | unknown | 140 |
| Insecta | Hymenoptera | Formicidae | unknown | 892 |
| Insecta | Lepidoptera | Geometridae | *Ectropis grisescens* | 465 |
| Insecta | Lepidoptera | Geometridae | *Scopula subpunctaria* | 827 |
| Insecta | Lepidoptera | Geometridae | unknown | 8 |
| Insecta | Lepidoptera | Limacodidae | unknown | 1 |
| Insecta | Lepidoptera | Lymantriidae | unknown | 48 |
| Insecta | Lepidoptera | unknown | unknown | 250 |
| Insecta | Orthoptera | Acrididae | unknown | 35 |
| Insecta | Orthoptera | Tettigoniidae | unknown | 222 |
| Insecta | Orthoptera | Trigonidiidae | *Paratrigonidium* sp. | 244 |
| Insecta | Thysanoptera | Thripidae | *Dendrothrips minowai* | 25 |
| Insecta | Thysanoptera | Thripidae | unknown | 585 |

**Table S5** Sequence statistics after the sequences were merged and filtered and chimeras were removed

|  | Sample ID | Number of sequence | | | |
| --- | --- | --- | --- | --- | --- |
|  |  | Raw | Merged | Filtered | Non-chimeric |
|  | COB_01 | 78725 | 76571 | 75235 | 75173 |
|  | COB_02 | 109001 | 106052 | 103460 | 103415 |
|  | COB_03 | 114705 | 111507 | 108676 | 108656 |
|  | COB_04 | 95103 | 92586 | 90466 | 90431 |
|  | COB_05 | 97485 | 95068 | 93239 | 93212 |
|  | COB_06 | 97063 | 94551 | 92641 | 92544 |
|  | COB_07 | 104816 | 102037 | 99980 | 99934 |
|  | COB_08 | 115811 | 112740 | 110012 | 109949 |
|  | COB_09 | 96088 | 93482 | 91367 | 91325 |
|  | COB_10 | 109985 | 106992 | 104669 | 104619 |
|  | COB_11 | 114017 | 110727 | 108019 | 107990 |
|  | COB_12 | 85067 | 82672 | 80554 | 80471 |
|  | COB_13 | 97486 | 94733 | 92422 | 92375 |
|  | COB_14 | 100250 | 97557 | 95170 | 95152 |
|  | COB_15 | 97208 | 94620 | 92406 | 92369 |
|  | COB_16 | 116044 | 113098 | 110812 | 110770 |
|  | COB_17 | 109885 | 106978 | 104624 | 104590 |
|  | COB_18 | 100502 | 97503 | 95496 | 95403 |
|  | COB_19 | 100266 | 97812 | 95934 | 95879 |
|  | COB_20 | 97370 | 94816 | 92559 | 92505 |
|  | COB_21 | 114813 | 111579 | 109208 | 109063 |
|  | COB_22 | 94540 | 92017 | 90039 | 89930 |
|  | COB_23 | 116768 | 113510 | 110829 | 110750 |
|  | COB_24 | 111057 | 108023 | 105794 | 105722 |
|  | COB_25 | 68672 | 62433 | 61364 | 61314 |
|  | COB_26 | 108463 | 104189 | 101825 | 100778 |
|  | COB_27 | 62858 | 62253 | 61044 | 61026 |
|  | COB_28 | 63932 | 63303 | 62192 | 62165 |
|  | COB_29 | 69153 | 68482 | 67477 | 67306 |
|  | COB_30 | 56724 | 56158 | 55290 | 55177 |
| Total |  | 2903857 | 2824049 | 2762803 | 2759993 |

**Table S6** Prey spectra of *C. blandum*

| Class | Order | Family | Genus | Species | Number of sequence |
| --- | --- | --- | --- | --- | --- |
| Arachnida | Araneae | Leptonetidae | *Neoleptoneta* |  | 1 |
| Arachnida | Araneae | Lycosidae | *Pardosa* |  | 5 |
| Arachnida | Araneae | Theridiidae |  |  | 30 |
| Arachnida | Araneae | Theridiidae | *Theridion* |  | 7 |
| Arachnida | Sarcoptiformes | Acaridae | *Tyrophagus* | *Tyrophagus putrescentiae* | 3 |
| Arachnida | Sarcoptiformes | Psoroptoididae | *Picalgoides* |  | 4766 |
| Arachnida | Trombidiformes | Cunaxidae |  |  | 3 |
| Arachnida | Trombidiformes | Eriophyidae | *Acaphylla* | *Acaphylla theavagrans* | 7 |
| Arachnida | Trombidiformes | Tarsonemidae | *Polyphagotarsonemus* |  | 2 |
| Chilopoda | Lithobiomorpha | Lithobiidae | *Eupolybothrus* |  | 11 |
| Chilopoda | Lithobiomorpha | Lithobiidae |  |  | 51 |
| Collembola | Entomobryomorpha | Entomobryidae | *Homidia* | *Homidia sinensis* | 7 |
| Insecta | Coleoptera | Chrysomelidae | *Gonioctena* |  | 13 |
| Insecta | Coleoptera | Cleridae | *Clerus* |  | 118 |
| Insecta | Coleoptera | Coccinellidae | *Harmonia* | *Harmonia axyridis* | 24 |
| Insecta | Coleoptera | Dermestidae |  |  | 5 |
| Insecta | Coleoptera | Elateridae | *Selatosomus* | *Selatosomus latus* | 43 |
| Insecta | Coleoptera | Scarabaeidae | *Anomala* |  | 24 |
| Insecta | Diptera |  |  |  | 3 |
| Insecta | Diptera | Asilidae | *Stenopogon* |  | 17 |
| Insecta | Diptera | Ceratopogonidae |  |  | 7 |
| Insecta | Diptera | Ceratopogonidae | *Culicoides* | *Culicoides oxystoma* | 4 |
| Insecta | Diptera | Chironomidae | *Tanytarsus* | *Tanytarsus formosanus* | 35 |
| Insecta | Diptera | Chironomidae | *Chironomus* | *Chironomus kiiensis* | 2 |
| Insecta | Diptera | Culicidae |  |  | 2 |
| Insecta | Diptera | Dolichopodidae | *Amblypsilopus* |  | 430 |
| Insecta | Diptera | Mycetophilidae |  |  | 3 |
| Insecta | Diptera | Tephritidae | *Zeugodacus* | *Zeugodacus cucurbitae* | 2 |
| Insecta | Diptera | Tipulidae |  |  | 87 |
| Insecta | Hemiptera | Aleyrodidae | *Aleurocanthus* | *Aleurocanthus spiniferus* | 1589 |
| Insecta | Hemiptera | Cicadellidae | *Empoasca* | *Empoasca onukii* | 14 |
| Insecta | Hemiptera | Clastopteridae |  |  | 109 |
| Insecta | Hemiptera | Fulgoridae | *Lycorma* |  | 251 |
| Insecta | Hymenoptera | Formicidae |  |  | 183 |
| Insecta | Hymenoptera | Formicidae | *Nylanderia* |  | 77 |
| Insecta | Lepidoptera | Cossidae | *Zeuzera* | *Zeuzera pyrina* | 68 |
| Insecta | Lepidoptera | Elachistidae | *Chlamydastis* |  | 5 |
| Insecta | Lepidoptera | Geometridae | *Ectropis* | *Ectropis grisescens* | 3 |
| Insecta | Orthoptera | Acrididae | *Acrida* | *Acrida cinerea* | 12 |
| Insecta | Orthoptera | Acrididae | *Chorthippus* |  | 41 |
| Insecta | Orthoptera | Acrididae | *Gomphocerus* |  | 63 |
| Insecta | Orthoptera | Acrididae | *Oedaleus* | *Oedaleus infernalis* | 213 |
